# The Semi‐Natural Climate Chambers across Latitudes: A Broadly Applicable Husbandry and Experimental System for Terrestrial Ectotherms under Climate Change

**DOI:** 10.1002/advs.202414185

**Published:** 2025-03-20

**Authors:** Bao‐Jun Sun, Hong‐Liang Lu, Kun‐Ming Cheng, Wan‐Li Liu, Xing‐Zhi Han, Luo‐Xin Cui, Xing‐Han Li, Shu‐Ran Li, Xin Hao, Fan Li, Dan‐Yang Wu, Teng Li, Yong‐Pu Zhang, Ji‐Chao Wang, Peng Liu, Wei‐Guo Du

**Affiliations:** ^1^ Key Laboratory of Animal Ecology and Conservation Biology Institute of Zoology Chinese Academy of Sciences Beijing 100101 China; ^2^ Hangzhou Key Laboratory of Animal Adaptation and Evolution Hangzhou Normal University Hangzhou 311121 China; ^3^ Ministry of Education Key Laboratory for Ecology of Tropical Islands College of Life Sciences Ministry of Education Hainan Normal University Haikou 571158 China; ^4^ College of Life Science and Technology Harbin Normal University Harbin 150025 China; ^5^ College of Life and Environmental Science Wenzhou University Wenzhou 325035 China; ^6^ School of Ecology Hainan University Haikou 570228 China; ^7^ College of Resources and Environmental Sciences Nanjing Agricultural University Nanjing 211800 China

**Keywords:** climate chamber, climate change, infrastructure, latitudes, terrestrial organism

## Abstract

With limited resources and efforts, assessing species' vulnerabilities across various geographic regions before the conservation practice is essential for biodiversity conservation in the context of climate change. One pressing challenge has been establishing natural temperature‐manipulated research systems across latitudes. To address this challenge, an innovative infrastructure is developed named the semi‐natural climate chambers across latitudes (SCCAL), consisting of semi‐natural climate chambers at three latitudes, spanning 27° and 3393 km from tropical to temperate regions. Each latitude features eight medium‐sized patches for temperature manipulation, organisms rearing, and ecological experiments. Independent of external water and electricity supplies, the SCCAL allows to simulate thermal environments under different climate change scenarios with natural soil moisture. Ecological experiments with Grass lizards successfully are conducted, demonstrating that the SCCAL effectively supports species rearing, responses determining, and the vulnerability assessing. The widespread adoption or development of similar infrastructures is encouraged, which can facilitate the assessment of latitudinal animal vulnerabilities under climate change.

## Graphical abstract

Assessing species' vulnerabilities across geographical gradients is essential for biodiversity conservation under climate change. To address this challenge, the semi‐natural climate chamber across latitudes (SCCAL) is developed, a validated system capable of simulating thermal environments for ecological experiments without reliance on water or electricity. The widespread adoption of SCCAL is advocated to facilitate ecological research on climate change.

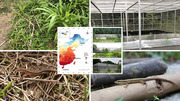

## 1 Introduction

Anthropogenic climate change poses a significant threat to global faunal biodiversity.^[^ ^1^, ^2^, ^3^ ^]^ However, the resources available for biodiversity conservation are limited, identifying species and locations with greater vulnerabilities is essential for implementing conservation strategies to fight against biodiversity loss. A current priority in conservation research is to assess the vulnerability of species across diverse geographic regions to climate change.^[^ ^4^, ^5^, ^6^, ^7^, ^8^ ^]^ Therefore, assessing the fitness‐related consequences of climate change on animals from different latitudes, along with comprehensively understanding the underlying behavioral and physiological mechanisms, are crucial not only to enhancing our understanding of environmental adaptation but also to identifying the geographic vulnerabilities of species in response to ongoing climate change.^[^ ^5^, ^9^, ^10^, ^11^, ^12^ ^]^

However, the latitudinal pattern of animal vulnerabilities to climate change remains contentious, and the understanding of it has “evolved” due to increasing parameters taken into considerations.^[^ ^12^, ^13^, ^14^, ^15^, ^16^ ^]^ Including more parameters generally leads toward a more comprehensive pattern.^[^ ^5^, ^17^, ^18^, ^19^, ^20^ ^]^ Nevertheless, obtaining a reliable latitudinal pattern experimentally presents significant challenges and obstacles. For instance, current research on geographic patterns of animal responses and vulnerabilities primarily relies on meta‐analysis, which aggregates data from various experiments with different designs and objectives.^[^ ^4^, ^14^, ^21^ ^]^ The experimental studies specifically designed to uncover latitudinal patterns of animal responses to climate change are still largely scarce.^[^ ^5^, ^22^ ^]^ Although sporadic experimental studies have established connections between animal responses and vulnerabilities to climate change, these are mostly conducted by comparison of natural populations without experimental manipulation,^[^ ^11^, ^21^, ^23^ ^]^ or by environment‐controlled indoor laboratories.^[^ ^12^ ^]^ Despite indoor experiments controlled average temperature and fluctuations, they are always weak in ecological‐complexity simulation.^[^ ^11^, ^12^ ^]^ Therefore, there is a critical need for the natural experimental arena that facilitates the rearing and direct observation of their behavioral and physiological responses across a broad latitude span under ecological conditions that can be changed.

Semi‐natural mesocosms have recently emerged as favored systems for researching the behavioral and physiological responses of terrestrial organisms in a more naturalistic setting.^[^ ^24^, ^25^, ^26^, ^27^, ^28^ ^]^ One of the most advanced systems is the “Metatron.”^[^ ^25^ ^]^ However, this system relies heavily on electricity and water resources, and its temperature in present‐ and simulated‐warming climates are not changed synchronously at daily cycle, which lacks ecological relevance.^[^ ^25^ ^]^ Moreover, a significant limitation of these semi‐natural mesocosms is their local scope, which often lacks a latitudinal gradient. Several factors induce this limitation. Firstly, these mesocosms are typically supported by the government or university and may not be able to cover a broad geographical range if the country or state is not sufficiently large.^[^ ^25^, ^29^ ^]^ Second, as these systems are designed for rearing and experimental purposes, they are often limited to species that are not widely distributed across large latitudinal ranges.^[^ ^30^ ^]^ Therefore, there remains an urgent need for a natural climate‐chamber arena capable of simulating climate change across a broad latitude while minimizing dependence on supplementary resources.

In 2018, we initiated the development of the semi‐natural climate chambers across latitudes (SCCAL): a resource‐efficient experimental infrastructure designed to span a significant latitudinal range and to facilitate environmental manipulation in ecological‐complexity simulations. The SCCAL comprises semi‐natural climate chambers situated across different latitudinal regions. The SCCAL accommodates a wide range of terrestrial organisms, from soil microbiota to small‐size vertebrates, and it is designed for rearing study species, conducting behavioral and physiological measurements, and assessing fitness consequences. It enables experimental manipulation of climate factors in ecological‐complexity simulations, addressing the urgent need for an outdoor study arena that can simulate climate change across a broad latitudinal range under natural conditions. The SCCAL was established with the support of the National Key Research Development Program of China and the National Natural Science Foundation of China, under the collaborative supervision of the Institute of Zoology (Chinese Academy of Sciences), Hainan Normal University, Hangzhou Normal University, and Harbin Normal University. This infrastructure system will advance research in behavioral and physiological ecology, and local adaptation in small animals, as well as contribute significantly to biodiversity‐conservation research under climate change.

## 2 Results

### 2.1 The Design and Assembly of the SCCAL

**Figure 1:**
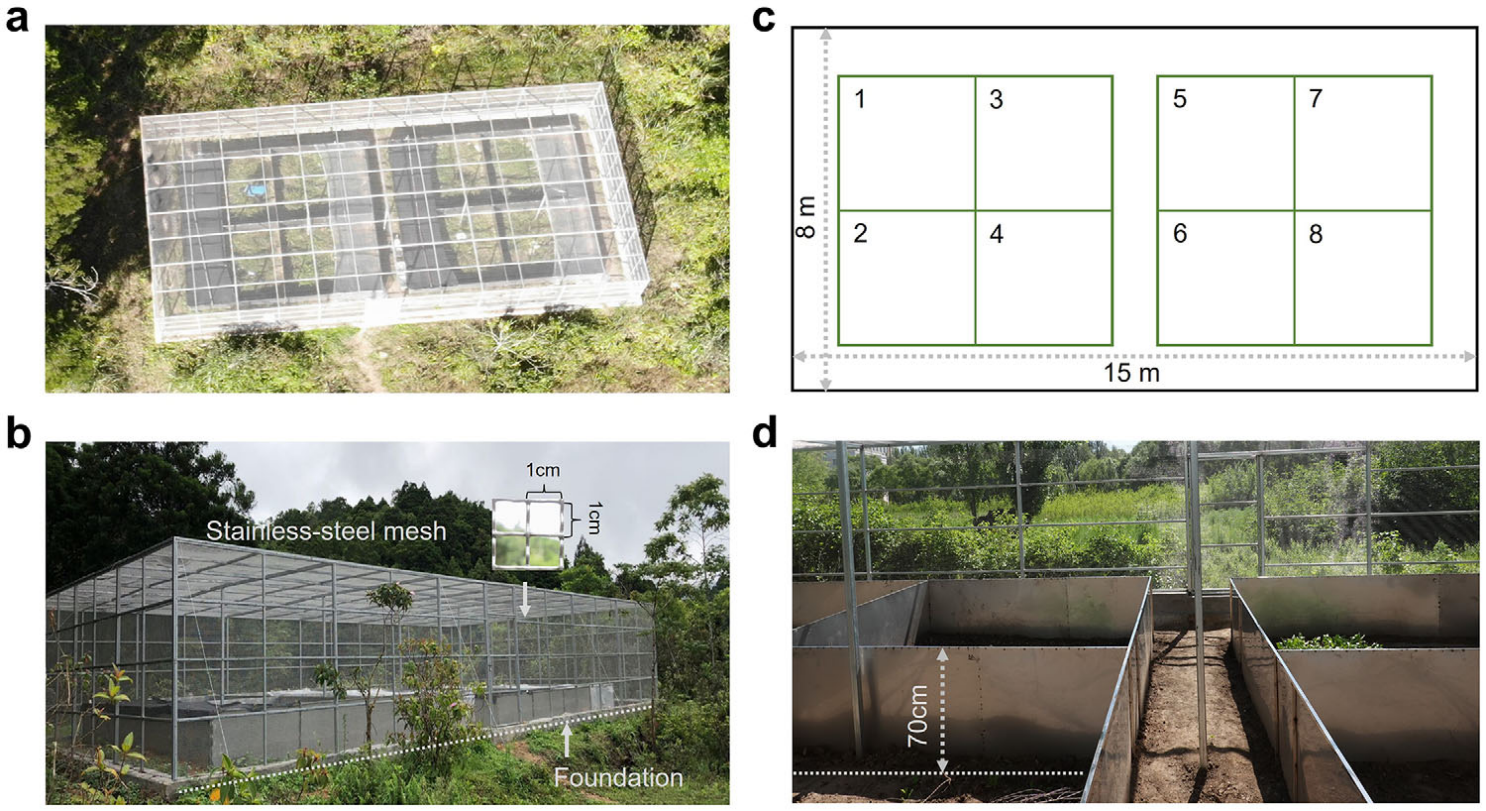
The design and assembly of the SCCAL. a) Aerial photo of the chamber at low latitude. The photo was taken in October 2019. b) the foundation and stainless‐steel mesh of the chamber. The photo was taken at low latitude in Jun 2019. c) Arrangement of eight patches inner each chamber. d) The inner view of patch and the above‐ground height of the stainless‐steel panels. The photo was taken in May 2020 at high latitude.

We developed a system named SCCAL, which is the acronym for semi‐natural climate chambers across latitudes. The SCCAL currently comprises three climate chamber systems at different latitudes in China: high latitude (temperate region at 45.87°N, 126.56°E, elevation of 116 m), medium latitude (subtropical region at 30.32°N, 120.40°E, elevation of 34 m), and low latitude (tropical region at 18.66°N, 109.93°E, elevation of 674 m). These chambers collectively cover a span of 27.2° in latitude, and 3393 km in distance (Figure S1, Supporting Information). At each latitude, the climate chamber covers an area of 120 m^2^, with an above‐ground volume of 288 m^3^ (15 m × 8 m × 2.4 m; length × width × height) (**Figure** **1**a) (Supporting Information 2). The perimeter of each chamber consists of foundations and stainless‐steel mesh. The foundation is built from brick and concrete, extending 0.3 m underground and 0.3 m above ground, to support the structure of steel frame and stainless‐steel mesh. The stainless‐steel mesh is demountable, with grids of 0.01 m × 0.01 m (Figure 1b). Inside each chamber, there are eight medium‐sized patches (3m × 3m ), each embraced by stainless‐steel panels (Figure 1c). The panels are 1.2 m in height, with 0.5 m embedded into the soil and 0.7 m above ground to effectively enclose the study organisms (Figure 1d) (see details in Supporting Information 2 for assembling of each chamber).

### 2.2 Principle of Operation

The combination of brick, concrete, and demountable stainless‐steel mesh enables effective management of the biotic factors within each chamber. The stainless‐steel mesh serves to prevent interference from avian predators, while the 0.3 m deep foundation of brick and concrete, along with the 0.5 m deep panel, protects against smaller predators such as rodents. Meanwhile, the buried 0.5 m stainless‐steel panel ensures that the study species are contained within each patch and are not able to escape through underground routes. The mesh can also exclude insects with larger body sizes than 0.01 m × 0.01 m. Therefore, the setup allows for precise control over predator presence and the majority of food availability within each patch.

**Figure 2:**
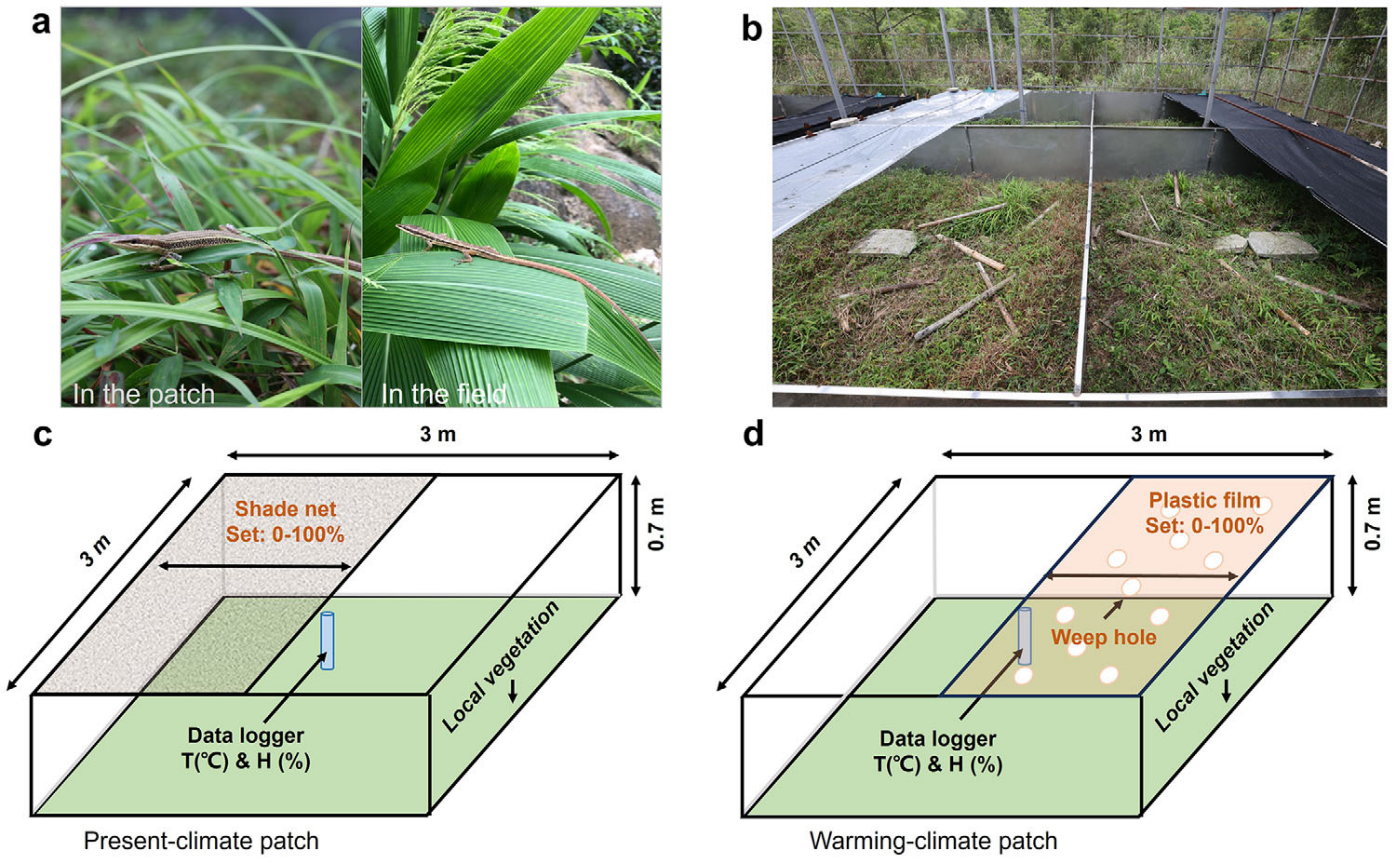
The principle of operation of the SCCAL. a) The vegetation and lizard of *T. kuehnei* in the patch and in the field at low latitude, respectively. b) The photo of shade net and plastic film set. c) The setup for the present‐climate patch. d) The setup for the warming‐climate patch. The area of shade nets and plastic films can be changed from 0% to 100%, to regulate the radiation and thus the temperatures in the patches. The local vegetations in the patches are transplanted from the natural microhabitats of the study organisms. Data loggers are set in the center of the patches.

In the SCCAL, abiotic environmental factors including illuminance, temperature, and soil moisture can be ecologically manipulated for each patch separately, according to the experimental design, without additional supply of electricity and water resources. Specifically, each patch can be equipped with vegetation that simulates natural microhabitats for study organisms by transplanting (**Figure** **2**a). The primary objective of the SCCAL currently is to simulate the thermal environment under varying climate change scenarios. Consequently, temperatures can be adjusted to reflect either present climate or warming climate scenarios. The manipulations of temperature within each patch can be achieved by regulating the area of shade net or plastic film (Figure 2b). Specifically, we can simulate the present climate conditions for the patch by setting the shade net made of black woven nylon. The shade net simulates natural shade provided by vegetation in natural microhabitats, thereby facilitating behavioral thermoregulation for reared organisms (Figure 2c). In contrast, the warming climate conditions for the patch can be created by greenhouse effects using plastic films. To prevent alterations in soil moisture, we cut some weep holes on plastic film to allow precipitation (e.g., rain) to pass through. The number and size of weep holes can be adjusted to achieve the desired soil moisture levels (Figure 2d). The area covered by the shade net and plastic film for each patch can be regulated from 0% to 100%, allowing precise manipulation over the temperature for present‐climate and warming‐climate patches. In addition, at the center of each patch, data loggers can be placed to record temperature and air humidity. Some additional environmental sensors can be added if required, including illuminance and flow rate (Figure 2c,d and Figures S3 and S4, Supporting Information).

### 2.3 Validation of Environmental Factors Manipulation

**Figure 3:**
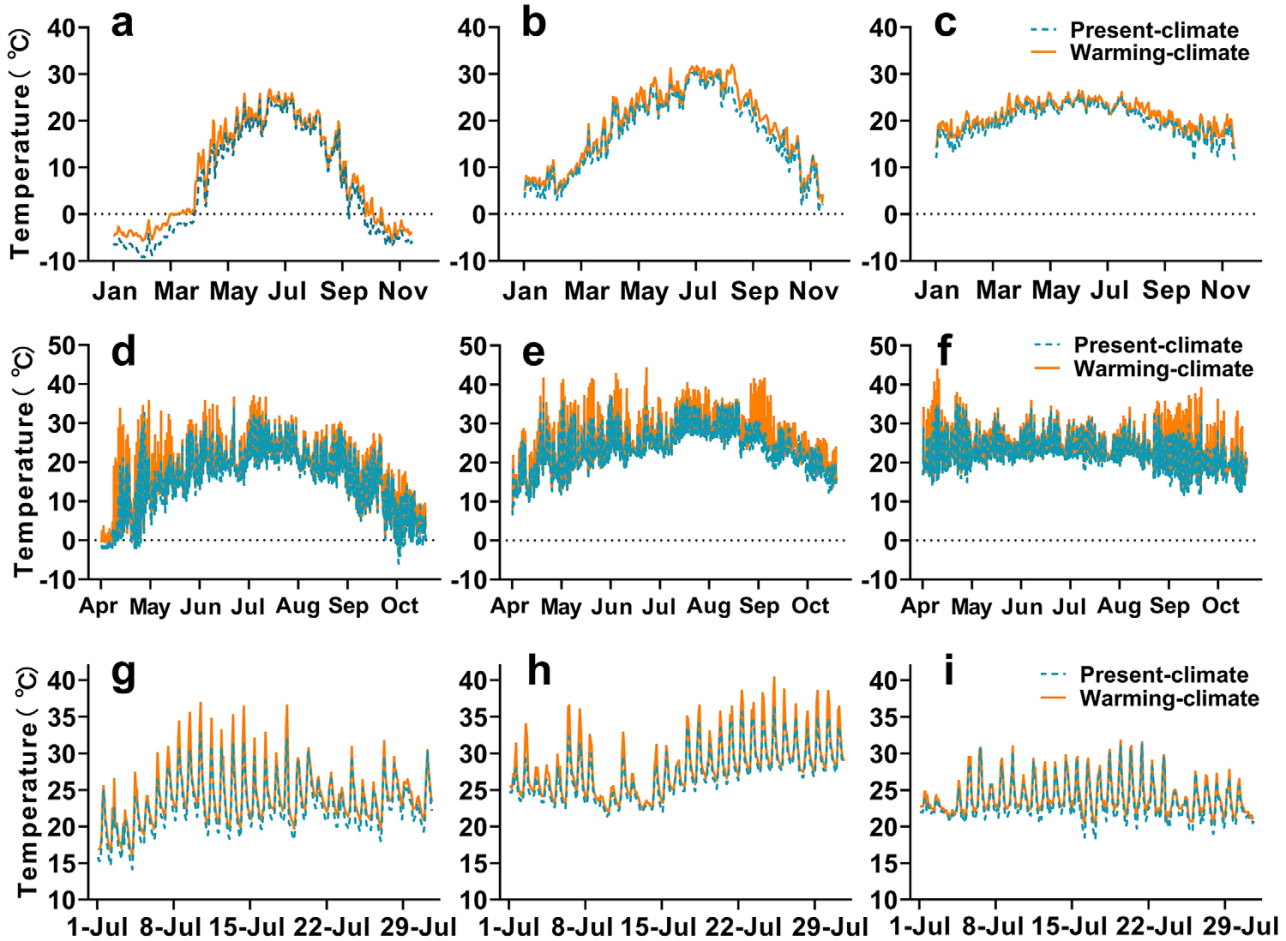
The average temperatures in present‐climate and warming‐climate patches through the year, active season, and hottest month. The temperatures at high latitude across a) the year, d) active season, and e) the hottest month. The temperatures at medium latitude across b) the year, e) active season, and h) the hottest month. The temperatures at low latitude across c) the year, f) active season, and i) the hottest month. The temperatures were collected every two hours. The green dashed and orange solid lines indicate the average temperature of four data loggers in the present‐climate and warming‐climate patches, respectively.

Following the establishment of the SCCAL, we conducted preliminary tests to validate the environmental setup for the patches (Figures S2–S4, Supporting Information). The results confirmed that the present‐climate patches replicated the thermal environments found in the natural microhabitats in ecology‐complexity simulations, as indicated by consistency in operative temperature (*T*_e_) between present‐climate patches and random locations in natural microhabitat outside the chamber (Figure S2, Supporting Information). The shade nets for present‐climate patches at each latitude are from 40%‐45% in area, in achieving this equality in average *T*_e_ between present‐climate patches and random locations in natural microhabitat. We further assessed the thermal environments across various temporal scales, including throughout the year, active seasons, and the hottest month, for both present‐ and warming‐climate patches. Our analysis revealed a significant difference in temperatures between the present‐climate and warming‐climate patches. Consistent with the prediction from IPCC that the warming rate increases towards higher latitudes,^[^ ^6^ ^]^ we have manipulated the temperature differences across the year at high (**Figure** **3**a,d,g), medium (Figure 3b,e,h), and low latitudes (Figure 3c,f,i) as 2.06 ± 0.06 °C, 1.73 ± 0.07 °C, and 1.30 ± 0.05 °C, respectively (**Table** **1**). Meanwhile, these differences are also consistent with the scenarios of moderate warming (SSP 1–2.6, 1.3‐2.4 °C).^[^ ^6^ ^]^ Currently, to achieve these differences, the area of plastic films covered on warming‐climate patches are 40%‐50% at different latitudes.

**Table 1:** Description of daily average temperature in present‐ and warming‐climate patches across latitudes. The data are shown as mean values, S.E., ranges, and statistical results. *T*_mean_ indicates daily average temperatures across the year; *T*_range_ (min‐max) indicates the minimum and maximum temperature across the year; △*T* indicates the difference between the average temperatures of present‐climate and warming‐climate patches. Significant results are shown in bold font.

	*T*_mean_ [°C]	S.E.	*T*_range_ (min‐max) [°C]	△*T* [°C]	**Statistical results**
Warming at high latitude	8.539	0.562	−5.672 – 26.675	2.06 ± 0.06	***t* = 36.54, *df* = 364,** ***P* < 0.001**
Present at high latitude	6.476	0.594	−9.250 – 25.243		
Warming at medium latitude	18.810	0.444	2.650 – 31.819	1.73 ± 0.07	***t* = 26.61, *df* = 364,** ***P* < 0.001**
Present at medium latitude	17.077	0.435	0.347 – 30.389		
Warming at low latitude	21.213	0.165	11.308 – 25.813	1.30 ± 0.05	***t* = 26.59, *df* = 364,** ***P* < 0.001**
Present at low latitude	19.909	0.146	13.917 – 26.375		

**Figure 4:**
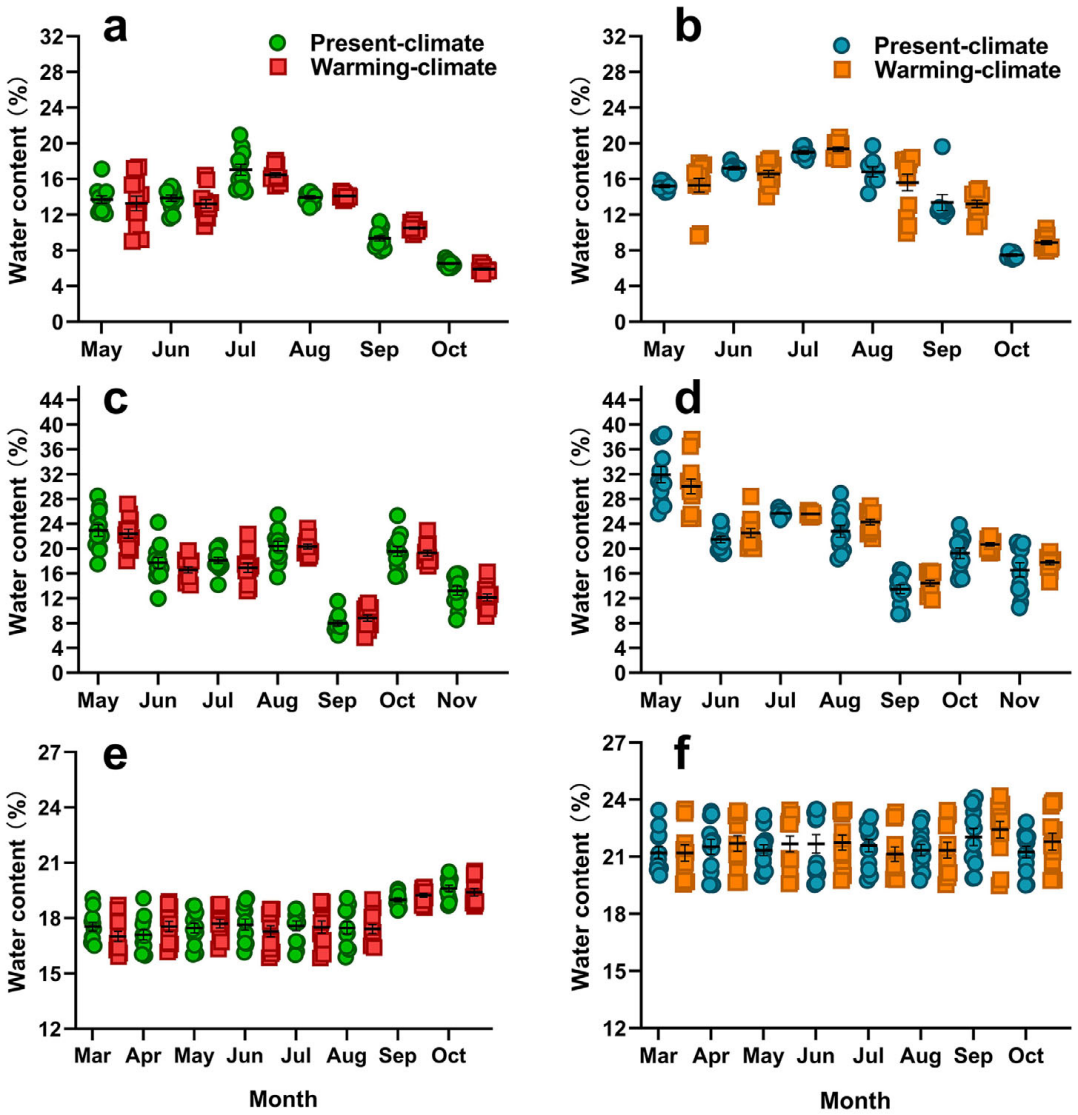
The moisture (water content) of soil samples collected from present‐climate and warming‐climate patches. The soil moisture on sunny days at a) high, c) medium, and e) low latitudes are expressed with green (present) and red spots (warming), while those on rainy days at b) high, d) medium, and f) low latitudes are expressed with blue (present) and orange spots (warming), respectively. Each spot indicates a value of a single soil sample; the data are shown as mean ± S.E; the sample size for each climate condition is equally 12 per month.

We monitored the soil moisture (water content) throughout the active season. The current soil moisture for warming‐climate patches is achieved by cutting eight or nine holes (diameter is 0.05 m) on plastic film for each patch, while the soil moisture for each present‐climate patch is achieved by woven shade net (Figure 2b). The soil moisture was significantly higher on rainy days compared to sunny days across all latitudes: high (*F*_1,44_ = 177.91, *P* < 0.001) (**Figure** **4**a,b), medium (*F*_1,44_ = 404.01, *P* < 0.001) (Figure 4c,d), and low latitudes (*F*_1,44_ = 937.53, *P* < 0.001) (Figure 4e,f). However, irrespective of weather conditions, the temperature manipulations by shade net and plastic film do not influence the soil moisture across any latitude: high (*F*_1,44_ = 0.058, *P* = 0.811) (Figure 4a,b), medium (*F*_1,44_ = 0.030, *P* = 0.863) (Figure 4c,d), or low latitudes (*F*_1,44_ = 0.140, *P* = 0.710) (Figure 4e,f) (**Table** **2**).

**Table 2:** Description of average moisture (water content) of soil samples collected from present‐ and warming‐climate patches across latitudes. The soil moisture is shown as on sunny and rainy days, respectively. Sunny indicates the soil moisture determined by 12 samples collected on sunny days and rainy indicates the soil moisture determined by 12 samples collected on rainy days. SM_present_ and SM_warming_ indicate the soil moisture (water content) of present‐climate and warming‐climate patches, respectively. The data are shown as mean ± S.E.

	SM_present_ [%]	SM_warming_ [%]	Statistical results
Sunny day at high latitude	12.401 ± 0.381	12.241 ± 0.373	*F*_1,22_ = 0.562, *P* = 0.461
Rainy day at high latitude	14.578 ± 0.401	14.823 ± 0.390	*F*_1,22_ = 0.736, *P* = 0.400
Sunny day at medium latitude	17.138 ± 0.545	16.333 ± 0.499	*F*_1,22_ = 2.321, *P* = 0.142
Rainy day at medium latitude	21.592 ± 0.660	22.183 ± 0.540	*F*_1,22_ = 2.528, *P* = 0.126
Sunny day at low latitude	17.927 ± 0.120	17.888 ± 0.123	*F*_1,22_ = 0.083, *P* = 0.776
Rainy day at low latitude	21.502 ± 0.128	21.630 ± 0.147	*F*_1,22_ = 0.417, *P* = 0.525

The illuminances are the lowest for the locations under shade nets, and the highest for those on the open ground, with those under plastic films in between (*F*_3,36_ = 2057.8, *P* < 0.001) (Figure S3, Supporting Information). The illuminance for the open ground of present climate patches and warming climate patches are similar (*F*_1,18_ = 0.012, *P* = 0.915). The illuminance was time dependent, with increasing along time in the morning (8 a.m. to 1 p.m.) and decreasing along time in the afternoon (1 p.m. to 4 p.m.) (*F*_8,288_ = 2959.6, *P* < 0.001) (Figure S3, Supporting Information). The average air humidity was not different between present climate patches and warming climate patches (*t* = −0.353, *df* = 4217, *P* = 0.724) (Figure S4, Supporting Information).

### 2.4 Fitness Consequence, Behavioral, and Physiological Responses

After the SCCAL was established, we first conducted an experiment with the Heilongjiang grass lizard (*Takydromus amurensis*) at high‐latitude chamber. In the patches, we incubated eggs and reared hatchlings under simulated moderate warming scenario (SSP1‐2.6).^[^ ^31^ ^]^ After, we assessed behavioral and physiological responses, and subsequently identified the fitness consequences (i.e., vulnerabilities) of hatchlings. A validation of the climate chamber setup and the methodology for assessing animal responses was achieved by this local experiment.^[^ ^31^ ^]^ In addition, we are currently conducting more research in the SCCAL based on the setups and methods verified by this research.

Briefly, following the establishment of the high‐latitude chamber in 2019, we incubated 144 eggs from 26 females and reared hatchlings in patches with a 2 × 2 factor‐manipulation design. We determined the hatching success and incubation period of embryos, as well as initial body size, metabolic rate, and survival of hatchlings.^[^ ^31^ ^]^ During the reproductive season (Jun to Aug), the daily average temperatures for embryos were 1.55 °C higher in the warming‐climate patches (25.59, 22.83–27.98 °C) compared to the present‐climate patches (24.04, 21.15–25.96 °C).^[^ ^31^ ^]^ The hatching success was equal between the patches (88.89%, 64/72).^[^ ^31^ ^]^ However, incubation periods differed, averaging 40.1 (30–46) days for eggs in present‐climate patches and 31.7 (24–42) d for siblings in warming‐climate patches.^[^ ^31^ ^]^ From July to September, the daily average temperatures for hatchlings were 1.06 °C higher in the warming‐climate patches (20.51, 9.93–26.70 °C) than those in the present‐climate patches (19.45, 9.11–25.43 °C).^[^ ^31^ ^]^ Correspondingly, the active body temperatures of hatchlings were 1.73 °C higher in warming‐climate patches (25.85, 19.1–35.1 °C) than in present‐climate patches (24.12, 14.6–34.6 °C).^[^ ^31^ ^]^ The survival rates before winter of hatchlings were 14.06% (9/64) for the hatchlings in the present‐climate patches and 31.25% (20/64) in the warming‐climate patches.^[^ ^31^ ^]^

## 3 Discussion

Given the constraints of limited resources and efforts, identifying the most vulnerable areas and species under climate change is theoretically and practically essential in biodiversity conservation.^[^ ^4^, ^32^, ^33^, ^34^ ^]^ Although a few types of outdoor mesocosms have been validated for local^[^ ^25^, ^30^, ^35^ ^]^ and present climate research,^[^ ^29^, ^36^ ^]^ empirically assessing the vulnerabilities of animals under climate change across latitudes remains challenging due to the scarcity of appropriate research facilities capable of supporting ecological studies spanning broad latitudes.^[^ ^12^, ^37^ ^]^ The semi‐natural climate chambers across latitudes (SCCAL) represents an innovative infrastructure system that offers the opportunities to rear small‐size terrestrial organisms from various latitudes under simulated climate change scenarios (Figures 3 and 4). In response to the urgent need for comprehensive analysis, the SCCAL aims to elucidate the latitudinal pattern of animal vulnerabilities to climate change, by examining the behavioral and physiological responses of animals to various thermal environments using ecological manipulations.^[^ ^31^, ^38^ ^]^

The SCCAL has been documented to be robust, convenient, and easily established.^[^ ^31^, ^38^ ^]^ First, the SCCAL is designed for ease of assembly and operation. Its structure is straightforward, with common materials that are neither custom‐designed nor expensive (Supporting Information 2). The brickwork, stainless‐steel mesh, and panels are long‐lasting materials and are easily replaceable if necessary. The shade nets and plastic films used are environmentally friendly and require minimal replacement. Typically, we renewed the shade nets once a year, while the plastic films twice a year. The maintenance needs of the SCCAL are infrequent. Over five years since the initial climate chamber was established at low latitude (2019 to 2024), only a few fasteners were replaced in 2024. Second, from the preliminary tests and the results of published research,^[^ ^31^, ^38^, ^39^ ^]^ the effectiveness and convenience of environmental manipulations in temperature, soil moisture, illuminance and air humidity were validated (Figures 3 and 4 and Figures S3 and S4, Supporting Information). Notably, the operations of the chambers are independent of supplementary resources of water and electricity, which facilitates both the experimental manipulation of ecological conditions and the expansion of the SCCAL. This independence allows for the construction of the SCCAL in remote locations and natural habitats of study species (Figure 1a). The extensively latitudinal span of the SCCAL can also support semi‐natural experiments aimed at studying local adaptation or species responses across various environments.

The published results of *T. amurensis* from the SCCAL are ecologically relevant and robust.^[^ ^31^, ^38^, ^39^ ^]^ The hatching success of *T. amurensis* in the patches (88.89%) is equally well to or even exceeds the average hatching success of other *Takydromus* lizards (*T. wolteri*: 59.4%; *T. septentrionalis*: 78.5%; *T. kuehnei*: 68.5%; *T. sexlineatus*: 77.8%) and indoor incubation rates for *T. amurensis* (78.9%).^[^ ^40^ ^]^ This suggests that the incubation environments provided in the patches are suitable for embryonic developments. Additionally, the incubation periods observed in the present‐climate (40.1 d) and warming‐climate patches (31.7 d) fall within the ranges of incubation period under indoor constant temperature regimes (41.8 d at 24 °C to 27.6 d at 32 °C).^[^ ^23^ ^]^ This variability further supports the ecological relevance and suitability of the thermal manipulations in the patches. The survival rate of the hatchlings in the present‐climate (14.06%) and warming‐climate patches (31.25%) were also consistent with the average survival rates (less than 30%) reported for numerous lizards.^[^ ^41^ ^]^ Admittedly the survival rates indoor experiments are typically higher than 30%, the survival rates observed in the patches provide a more ecologically accurate reflection of lizard survival in natural conditions.^[^ ^31^, ^38^, ^39^, ^42^, ^43^ ^]^

The SCCAL can also accommodate a diverse category of terrestrial organisms, including soil‐dwelling microbiota, small‐size plants, invertebrates, flightless insects, and small vertebrates. For example, the SCCAL supports a diverse of invertebrate communities including earthworms, caterpillars, ants, spiders, crickets, and grasshoppers, providing the possibility for us to conduct research on these organisms. As demonstrated with other mesocosms, we can provide appropriate vegetation, retreats, and microhabitats tailored to specific organisms if needed.^[^ ^35^, ^44^, ^45^ ^]^ The SCCAL can also support research on dispersal insects, by incorporating insect‐proof nets in the patches and on dispersal lizards with some connective tunnels.^[^ ^44^ ^]^

Despite its advantages, the SCCAL does have some limitations that can be refined. One notable constraint is that the current setup of the SCCAL excludes the influence of predators, particularly avian predators. The exclusion of avian predators might lead to an over‐optimistic estimate of stress and survival rates.^[^ ^46^ ^]^ However, the stainless mesh of the SCCAL is demountable, allowing for the potential inclusion of predators if necessary. Another possible limitation is related to food availability. We supply supplementary crickets, and larva of *Tenebrio molitor* to the reared lizards, which may result in somewhat artificial control over food intake. Nonetheless, natural food resources for lizards such as spiders, crickets, and worms are not excluded from the patches during the experiments, ensuring that food abundance and amount are maintained for reared animals. A further consideration is the debate over whether the group of lizards in a patch truly represents a population. Although we reared the number of individuals based on field data, the patches constrain natural disperse, mortality, and recruitment.^[^ ^25^, ^47^ ^]^ Consequently, caution is warranted when extrapolating findings to the level of population dynamics. Nevertheless, the current configuration and manipulation of the SCCAL are appropriate for investigating behavioral and physiological responses, as well as fitness consequences such as reproduction and survival, as it is widely documented that the SCCAL can effectively reflect various organismal responses to environmental variations.^[^ ^29^, ^35^, ^36^, ^43^ ^]^ As the development of mathematic model and artificial intelligence (AI),^[^ ^48^ ^]^ population dynamics can be estimated and predicted based on individual responses such as reproduction, hatching success, and survival rate.^[^ ^49^, ^50^ ^]^ For instance, using the traits of individual growth rate, survival rate, and reproductive output, we can apply the Unified Life Model software to project the population dynamics.^[^ ^50^ ^]^ Some recently developed matrix models can also facilitate the assessment of population dynamic.^[^ ^48^ ^]^ Last, the SCCAL currently includes three unified chamber systems situated at high, medium, and low latitudes, which only meets the needs of the minimum number of gradients for the research on latitudinal patterns. We plan to expand this system by constructing two additional enclosures, creating a comprehensive network of five chambers with an average interval of 7° in latitude, and 800 km in distance. Furthermore, we inspire ecologists to participate in the development of semi‐natural climate chambers across a broader latitude. This will enhance our ability to estimate more solid latitudinal patterns of organismal vulnerabilities to climate change.^[^ ^12^, ^37^ ^]^ Also, we welcome collaborations with ecologists worldwide to utilize existing infrastructures for global research on animal vulnerability to climate change.

## 4 Conclusion

Climate change has imposed multiple threats to biodiversity, making it imperative to identify the most vulnerable areas and species. The SCCAL offers practical infrastructure and method to evaluate the vulnerabilities of organisms to climate change across latitudes,^[^ ^31^, ^38^ ^]^ with more ecological relevance in simulation and fewer supplementary resource requests. Utilizing the SCCAL, an innovative infrastructure, researchers can conceive rigorously ecological experiments to reveal the crucial latitudinal patterns and test innovative hypotheses related to climate change.

## 5 Experimental Section

### The Vegetation Equipped in the Patches

In 2018 and 2019, after the establishment of the chamber at each latitude, the vegetation was equipped for patches by transplanting to simulate the natural microhabitats of grass lizards, following published methods.^[^ ^31^, ^39^ ^]^ Specifically, local vegetation from lizard's natural microhabitats was collected in the field and transplanted into the patches. Subsequently, the vegetation was cultivated and maintained, adjusting its height to align with the typical activity level of the lizards (i.e., around 0.3 m). After two rounds of vegetation maintenance, the vegetation in the patches successfully replicated the natural microhabitat was considered. The thermal environments within the patches were then proceeded to manipulate.

### Validation of Thermal Environments Throughout the Year

From 2019, four patches at each latitude were designated to represent the present climate, while the other four patches simulated the warming climate.^[^ ^31^ ^]^ The present‐climate patches were partially covered with the shade nets, while the warming‐climate patches were partially covered with plastic films to retain radiant heat through the greenhouse effect. The coverage areas of the shade nets and plastic films were adjusted daily based on the temperature records on the previous day. In detail, data loggers were first placed in randomly selected natural microhabitats and present‐climate patches to record the *T*e hourly. Each night, the recorded data were reviewed and then the areas of the shade nets were adjusted to ensure that temperatures in present‐climate patches were equal to those in natural microhabitats. After the coverage of the shade nets in present‐climate patches were confirmed, the area of plastic films was proceeded to adjust for warming‐climate patches using the same approach. Data loggers were placed in both present‐ and warming‐climate patches to record the *T*e, and the plastic film coverage was adjusted daily based on the temperature record on the previous day. Once the temperature differences between the present‐ and waring‐climate patches aligned with the moderate warming scenario of SSP 1–2.6 (i.e., 1.3–2.4 °C),^[^ ^6^ ^]^ and these differences between present‐ and warming‐climate patches increased progressively toward high latitudes, the areas of both the shade nets and the plastic films were finalized.

Subsequently, monitoring the operative temperatures (*T*_e_) in the patches using data loggers (iButton, DS1921; MAXIM Integrated Products Ltd., CA, USA) enclosed in copper tube models with plasticine was begun. The copper tubes were designed to approximate the size of adult lizards (7 cm length × 2.5 cm diameter).^[^ ^43^ ^]^ Four data loggers in each climate condition were placed: two loggers were positioned under shade net for the present climate or under the plastic film for the warming climate, while the remaining two were placed on the open ground in each climate patch. The temperatures were recorded every 2 h. Data from the loggers were read every two months, and the loggers were replaced in the same positions throughout the study. The average temperature of four data loggers from present‐climate patches and warming‐climate patches as *T*_e_ for present climate and warming climate was calculated, respectively.

### Soil Moisture through the Active Season

Soil moisture was assessed monthly during the active season on both sunny and rainy days in 2020. Soil moisture measurements were taken over eight months (March to October) at low latitude, seven months (May to November) at medium latitude, and six months (May to October) at high latitude, respectively. In each month, 12 soil samples were collected from each of present climate and warming climate on each of sunny and rainy days, respectively. The locations of soil sampling were randomly selected under the shade net/plastic film and on open ground in the patches. For sunny‐day samples, soil was collected after a minimum of two consecutive sunny days. For rainy‐day samples, soil was collected at least 2 h after rainfall. Samples were collected randomly using a shovel from sites at a depth of 5 to 10 cm and immediately placed in sealed plastic bags. Each soil sample weighed between 300 and 500 g. Upon collection, soil samples were weighed immediately, and then transferred to an oven for drying. The drying was at 80 °C for 48 h. The water mass was calculated as the difference in weight before and after being dried, with the following formula: Soil moisture = 100% × water mass/wet soil mass. The average soil moisture of 12 samples was calculated as the soil moisture for each climate on each of sunny and rainy days.

### Validations of Illuminance and Air Humidity

In addition to temperature and soil moisture, the validations of illuminance and air humidity were conducted. The illuminance was quantified hourly from 8 a.m. to 4 p.m. only on a single day in early spring at the high‐latitude chamber, following the establishment of the SCCAL. This one‐time measurement was deemed sufficient due to the consistency of illuminance under clear‐sky conditions. An illuminometer was used to collect 10 measurements under shade nets and on open ground in present‐climate patches, as well as 10 measurements under plastic films and on open ground in the warming‐climate patches, respectively.

Air humidity was recorded hourly using four data loggers (TP2200, A‐volt, China) throughout the active season at high latitudes for validations. For both present‐ and warming‐climate patches, two loggers were placed at the center of patches at a height of 0.5 m (one logger per patch) to quantify air humidity. The average air humidity was then calculated for each climate condition.

### Routine Maintenance for the SCCAL

During ecological experiments in the SCCAL, routine maintenance was performed as needed, including cleaning, reinforcing, and replacing shade nets and plastic films, maintaining vegetation, and inspecting for invasive animals.

Specifically, a thorough inspection was conducted to ensure that the shade nets and plastic films were clean, secure, and undamaged. For cleaning, brooms were used for the shade nets and towels for the plastic films. The component was reinforced accordingly if it was found to be loose. In cases of damage, the affected shade nets or plastic films were replaced. Typically, shade nets (once per year) and plastic films (twice per year) were renewed at a low frequency.

To maintain a grass‐free zone around the patches, grass growing along the edges of the stainless‐steel panels was carefully trimmed or removed, keeping the area clear within approximately 0.05 m of the patch boundary. Additionally, vegetation inside the patches was trimmed every two weeks to maintain a consistent height of around 0.3 m.

Routine inspections were also conducted to identify potential threats to lizards from other animals, such as burrowing rodents and fire ants. If rodent burrows were discovered, we would immediately search for the rodents and promptly fill the burrows. In cases where fire ant nests were identified, we would prioritize recapturing any affected lizards before implementing ant eradication measures. Since the establishment of the SCCAL, we have not detected any other animals posing a threat to the lizards, such as burrowing rodent or fire ants.

### Statistical Analysis

All statistical analyses were conducted using STATISTICA software (version 10.0). A descriptive analysis of the temperatures and soil moisture was first performed. The mean, standard error, minimum, maximum and the difference in operative temperatures (*T*_e_) between present and warming climates at each latitude were calculated. Paired *t‐* *tests* were employed to detect the difference in *T*_e_ between the present and warming climates. Soil moisture was described as mean ± standard error (S.E.). To examine the differences in soil moisture between sunny days and rainy days, present and warming climates, one‐way ANOVAs were conducted. The illuminance was analyzed with repeat measures ANOVAs, with climate condition (i.e., present vs warming) and locations (i.e., under shade nets, open ground in present patch, under plastic films, open ground in warming patch) as main factors, and with time as repeat measures. The air humidity was analyzed by paired *t‐tests*. Prior to the analyses, we tested the normality of distributions and homogeneity of variances using *Kolmogorov‐Smirnov* and *Bartlett's* tests. All environmental data sets for analysis are deposited in the Science Data Bank^[51]^.

## Conflict of Interest

The authors declare no conflict of interest.

## Author Contributions

B.‐J.S., H.‐L.L., K.‐M.C., and W.‐L.L. contributed equally to this work. B.‐J.S. and W.‐G.D.: Lead contact. B.‐J.S. and W.‐G.D. convinced the initial ideas and managed the funds to support the establishment of the SCCAL. J.‐C.W. primarily manages the enclosure at low latitude, Y.‐P.Z. and H.‐L.L. primarily manages the enclosure at medium latitude, and P.L. primarily manages the enclosure at high latitude. B.‐J.S., H.‐L.L., J.‐C.W., P.L., and X.‐Z.H. participated in the final construction of the SCCAL. The data for environmental variations, lizard research projects, and soil microbiota were collected by K.‐M.C., W.‐L.L., L.‐X.C., X.‐H.L., S.‐R.L., X.H., F.L., D.‐Y.W., T.L., and Y.‐P.Z. The original draft and final editing of this paper were conducted by B.‐J.S. and H.‐L.L. All authors commented on and approved the final version of the paper.

## Supporting information



Supporting Information

## Data Availability

The environmental dataset of the SCCAL that support the findings of this study are openly available in Science Data Bank at https://doi.org/10.57760/sciencedb.20986, reference number 2098651.
