# Supplementary material for: The Semi‐Natural Climate Chambers across Latitudes: A Broadly Applicable Husbandry and Experimental System for Terrestrial Ectotherms under Climate Change
Source: Adv Sci (Weinh). 2025 Mar 20;12(20):2414185. doi: 10.1002/advs.202414185 (PMC12120752; doi:10.1002/advs.202414185)
Supplement: Supplementary file 1 — Supporting Information [file ADVS-12-2414185-s001.docx]

Supporting Information

The Semi-Natural Climate Chambers Across Latitudes: A Broadly Applicable Husbandry and Experimental System for Terrestrial Ectotherms under Climate Change

Bao-Jun Sun*, Hong-Liang Lu*, Kun-Ming Cheng, Wan-Li Liu, Xing-Zhi Han, Luo-Xin Cui, Xing-Han Li, Shu-Ran Li, Xin Hao, Fan Li, Dan-Yang Wu, Teng Li, Yong-Pu Zhang*, Ji-Chao Wang*, Peng Liu* and Wei-Guo Du*

**Supporting Information 1. The locations, validation of the settings in present-climate temperatures, illuminances and air humidities of patches.**


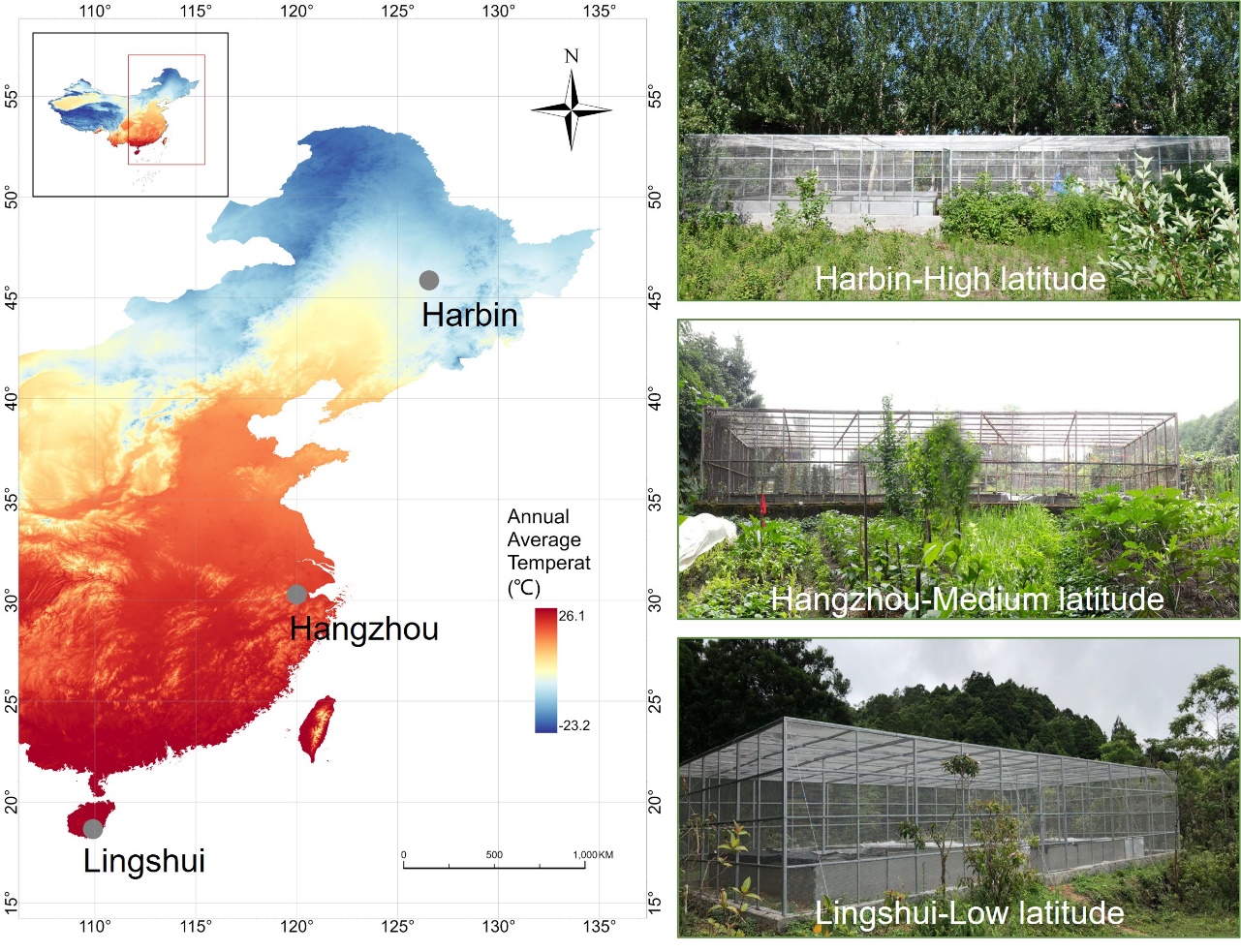


**Fig. S1. The locations for each chamber of the Semi-Natural Climate Chamber Across Latitudes (SCCAL).**

The SCCAL currently comprises three chambers at low- (Lingshui), medium-(Hangzhou), and high-latitude (Harbin), respectively. The gradient color from blue to red on the map indicates the increasing annual average temperature (℃). The design and assembly of each chamber are unified across latitudes.


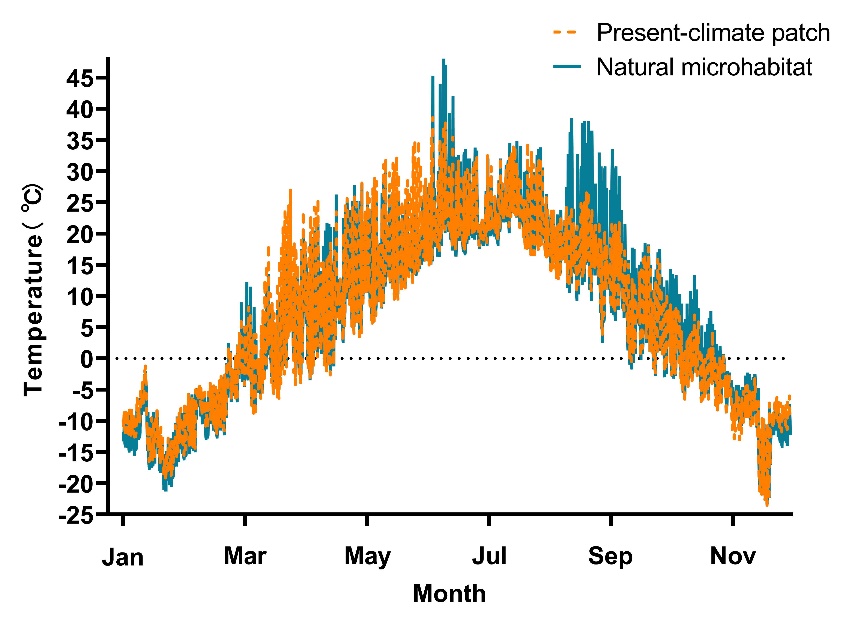


**Fig. S2. The temperatures of the present-climate patch and natural microhabitat at high latitude.**

The temperatures were collected every two hours across the year of 2021. The orange and blue lines indicate the average operative temperature (*T*_e_) for a present-climate patch and the average *T*_e_ for random locations in natural microhabitat outside the chamber. The average *T*_e_ was not significantly different between the present-climate patch (6.33 ± 0.20 ℃, Mean ± SE) and the natural microhabitat (6.22 ± 0.20 ℃, Mean ± SE) (*t*=0.3889, *df*=8746, *P*=0.697). This consistence of *T*_e_ indicates that the setting of the present-climate patch can successfully simulate the thermal environments in natural microhabitat ecologically.


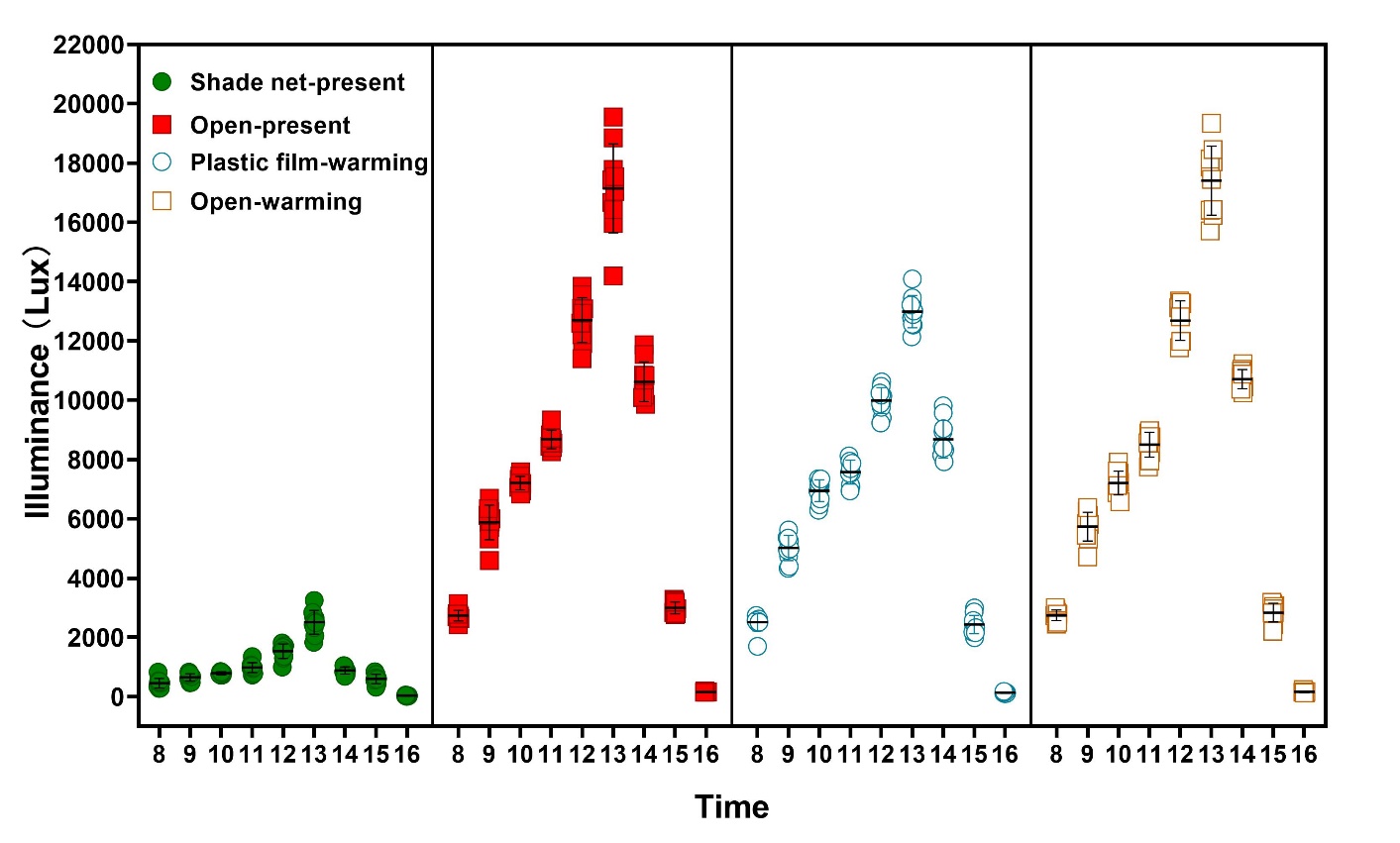


**Fig. S3. The illuminance (Lux) of the present-climate and warming-climate patches at high latitude.**

The illuminances were collected hourly. The green, red, blue and orange spots indicate the illuminance under shade net of present-climate patch, in the open area of present-climate patch, under plastic film of warming-climate patch, and open area of warming-climate patch, respectively. We repeatedly collected illuminance from ten random locations for each type of place across timescale, and each spot indicated one measurement of illuminance at one location.


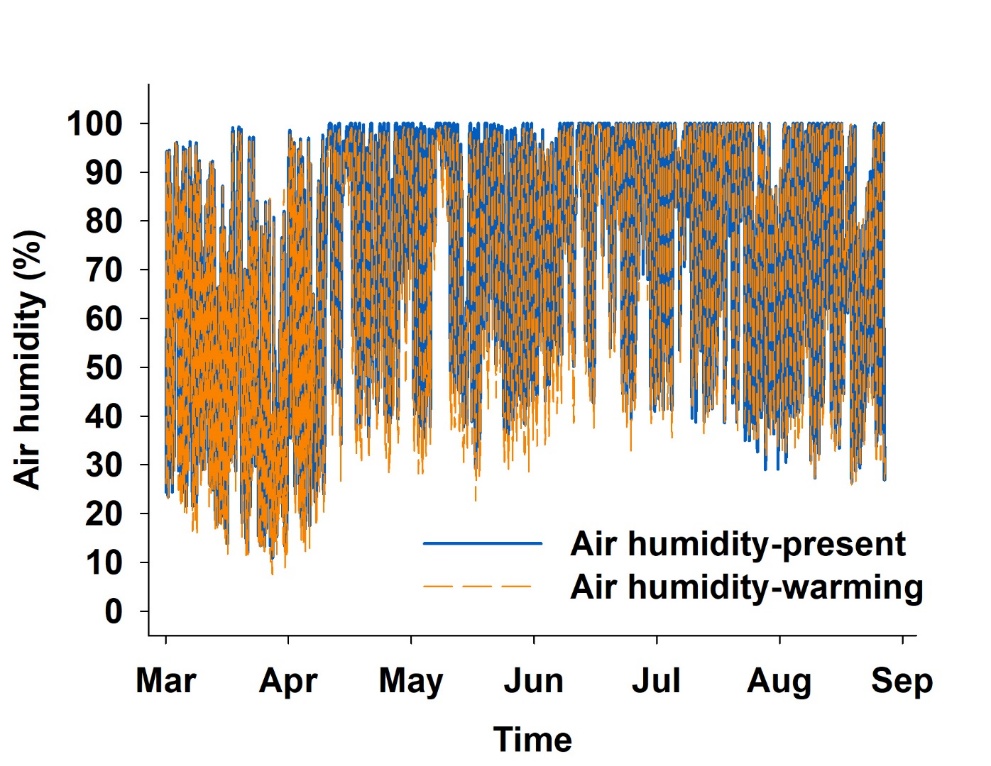


**Fig. S4. The air humidities of the present-climate and warming-climate patches at high latitude.**

The air humidities of patches were collected hourly by two data loggers for each climate. The blue and orange lines indicate the average air humidities in the present- and warming-climate patch, respectively. The data loggers were set in the center of the patch at a height of 0.5m.

**Supporting Information 2. Construction materials, assembling and additional photos for the Semi-natural Climate Chamber Across Latitudes (SCCAL)**

**1. Construction materials for the SCCAL at each latitude**

***Concrete Foundation*** The concrete foundation is rectangular and totally 46m (8m+8m+15m+15m) in length. For each side of foundation, the width is 40cm and the height is 60cm (30cm underground and 30cm above ground). A ring beam is burrowed underground within the concrete foundation to fix the constructure.

***Steel Frame*** The steel frame is made of 125 pieces of square steel. Each piece of square steel is 2.7m in length with a side length of 6cm. The thickness of the square steel is around 2mm.

***Stainless-steel Mesh*** Stainless-steel mesh is made of stainless-steel wire. The total area for the mesh is 420m2 and each mesh openings (grid) is square with a side length of 1cm.

***Stainless-steel Panel*** The patch is embraced by 1.2m-wide stainless-steel panel, and the total length of the panels is 72m. To hold the steel panels, 120 pieces of square stainless-steel columns are used. Each piece is 1.2m in length, with the side length is 3cm and thickness is 1mm.

***Door*** The SCCAL at each latitude has a door made of steel frame and stainless-mesh. The door is 1m in width and 1.8m in height.

In summary, to construct the SCCAL at each latitude, at least following materials are necessary: 11.5 m^3^ concrete (46m×0.4m×0.6m); 125 pieces of square steel (2.7m in length, 6cm in width); 420 m^2^ stainless-steel mesh; 72m stainless-steel panel (1.2m in width); 120 pieces of square stainless-steel columns (1.2m in length, 3cm in width); a door. Accordingly, SCCAL for each latitude cost around 140 thousand Yuan (CNY)(i.e., 20 thousand in US dollar)(Table S1).

**Table S1. Construction materials and related cost for the SCCAL at each latitude.** The cost is calculated as CNY.

| **Item** | **Unit** | **Quantity** | **Price/Unit** | **Sum (CNY)** |
| --- | --- | --- | --- | --- |
| Concrete | m^3^ | 11.5 | 700/m^3^ | 8050 |
| 6cm Square steel | piece | 125 | 220/piece | 27500 |
| Stainless-steel mesh | m^2^ | 420 | 50/m^2^ | 21000 |
| Stainless-steel panel | m | 72 | 300/m | 21600 |
| 3cm Square steel | piece | 120 | 70/piece | 8400 |
| Door | piece | 1 | 1000/piece | 1000 |
| Transportation |  |  | 2000 | 2000 |
| Labor cost |  |  | 50000 | 50000 |
| Total cost |  |  |  | **139550** |

**2. Assembling of materials for the SCCAL at each latitude**

***Side view-Perimeter1***


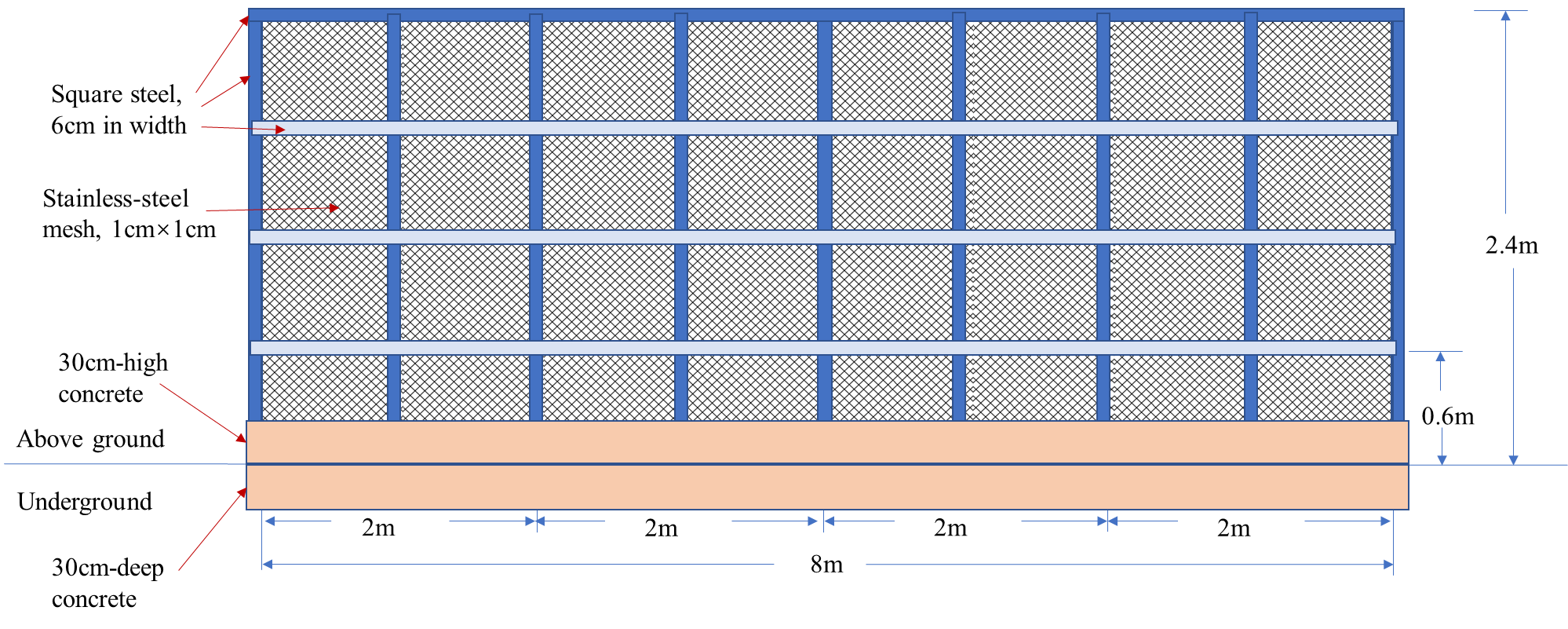


***Side view-Perimeter2***


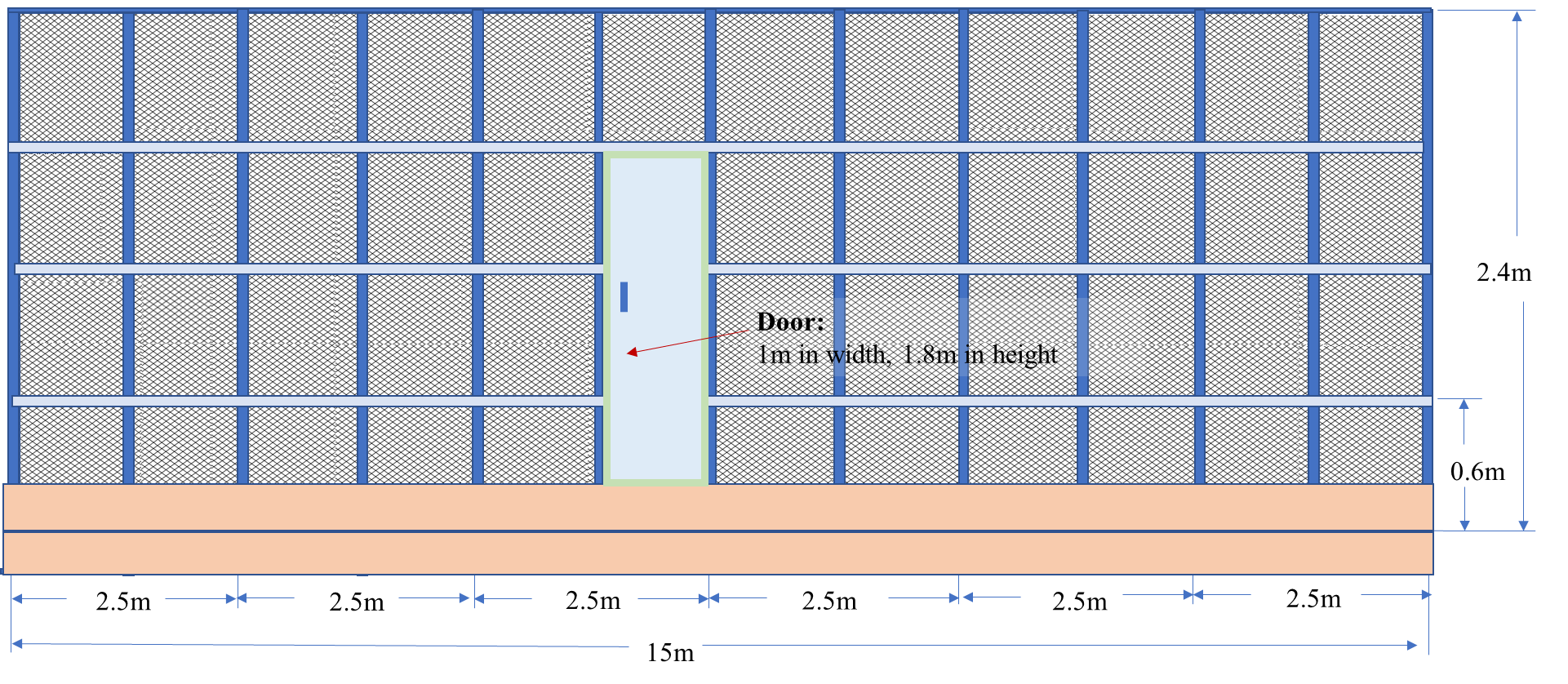


***Top view-Perimeter***


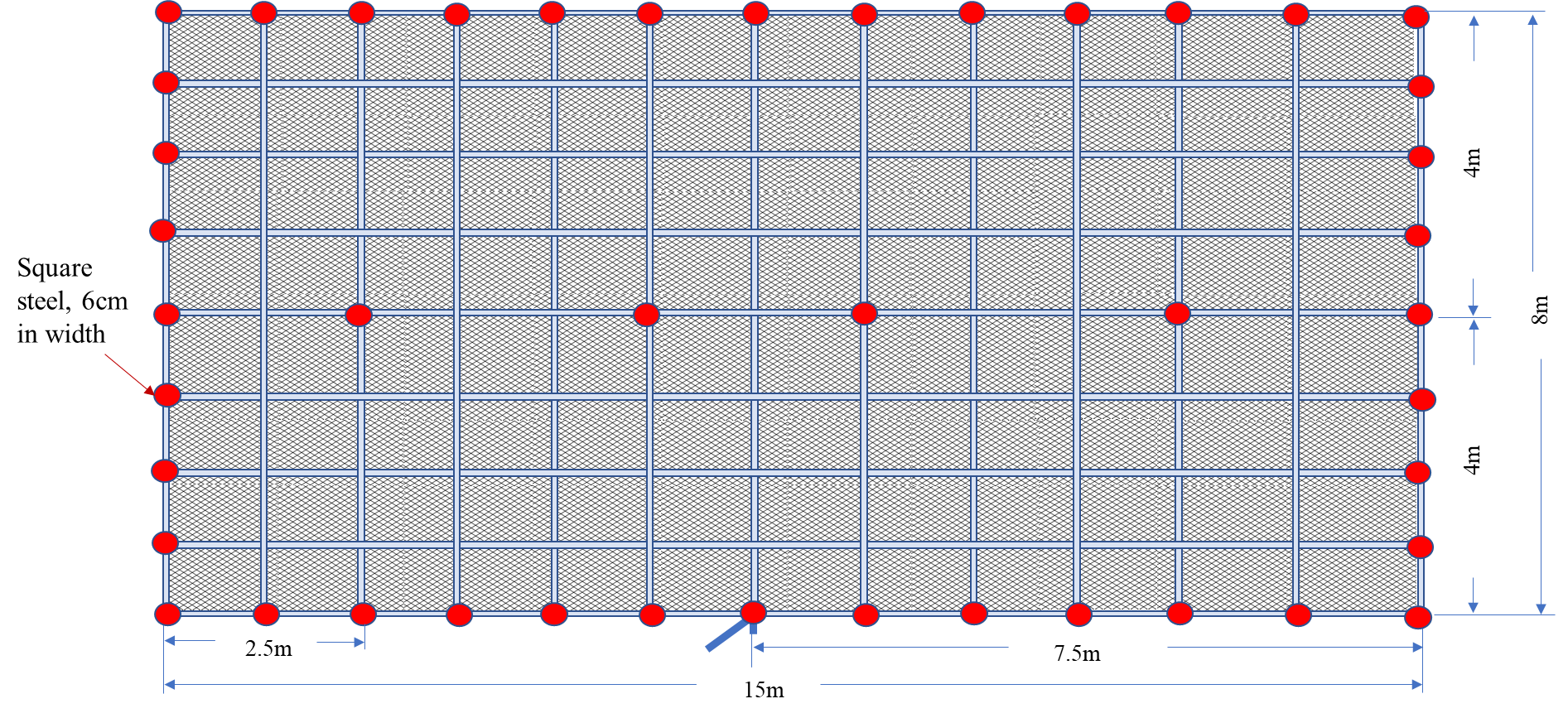


***Side view-Patch***


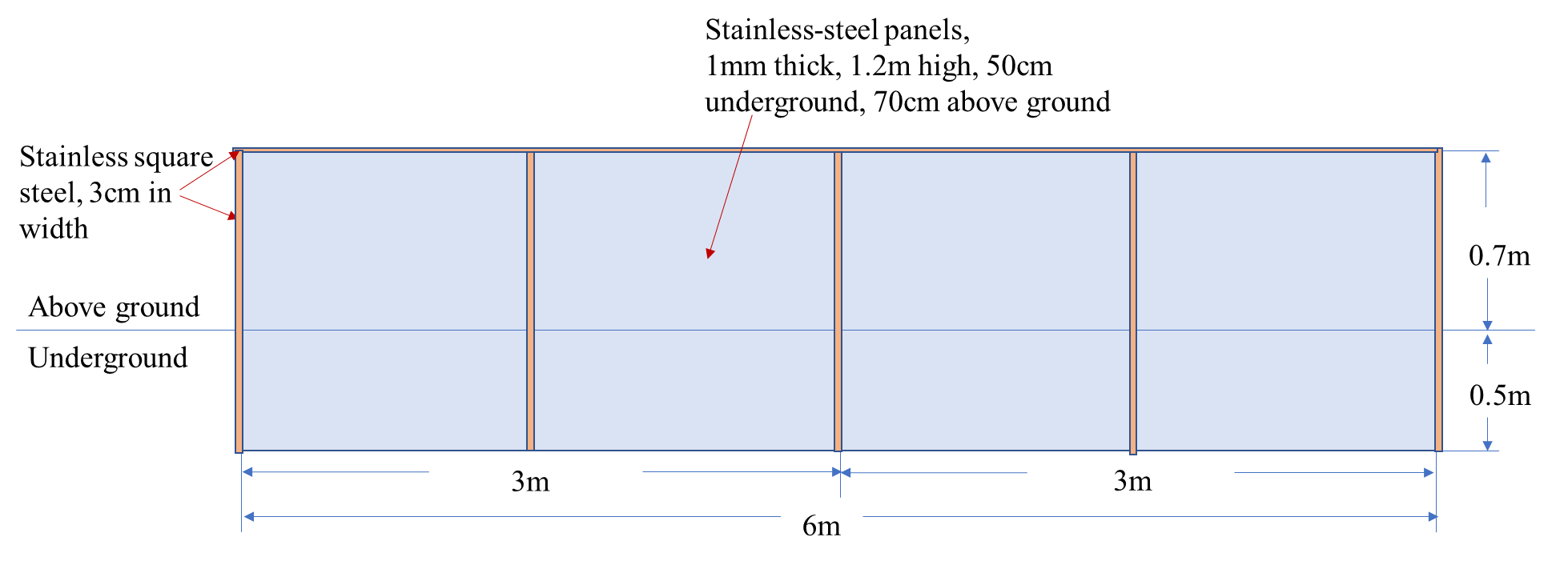


***Top view-Patch***

***
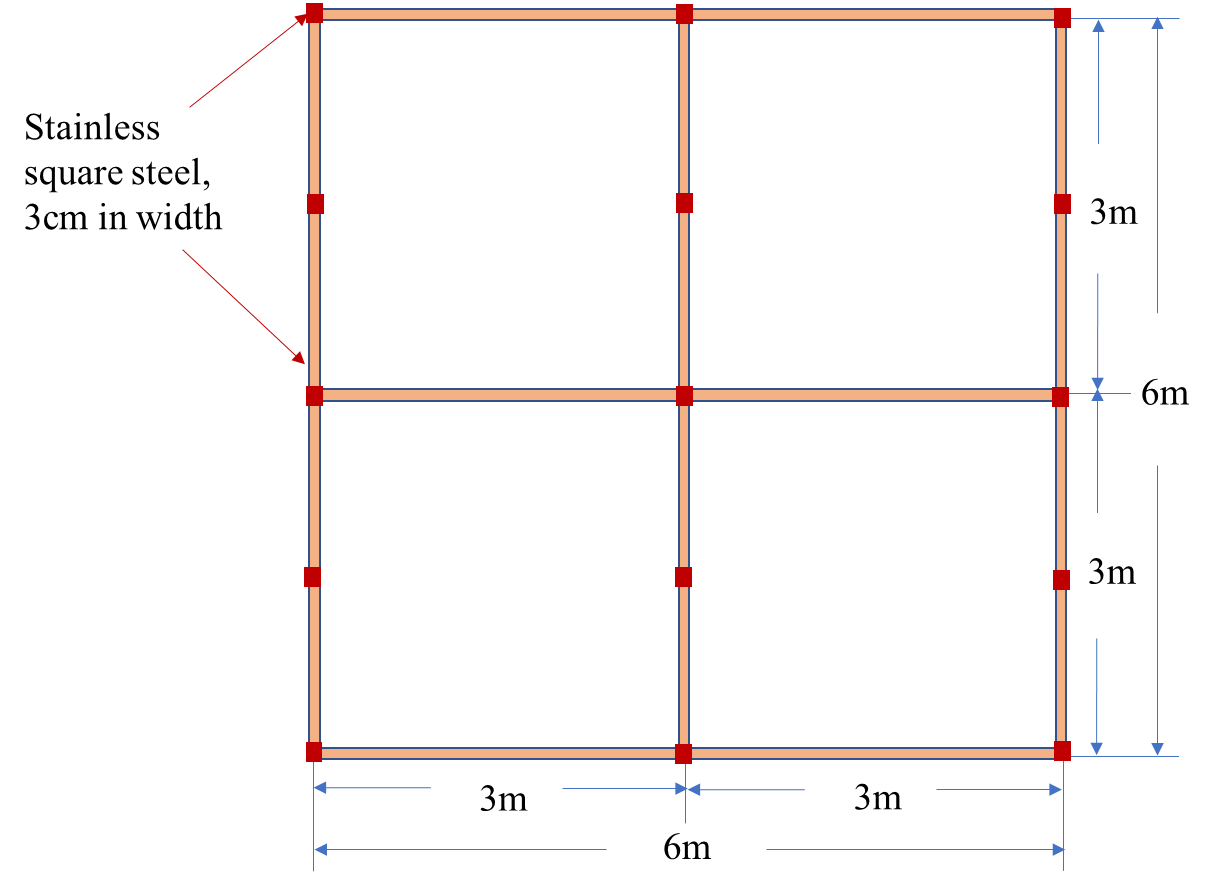
***

**3. Additional photos for the SCCAL**

***Side view-Perimeter***

*
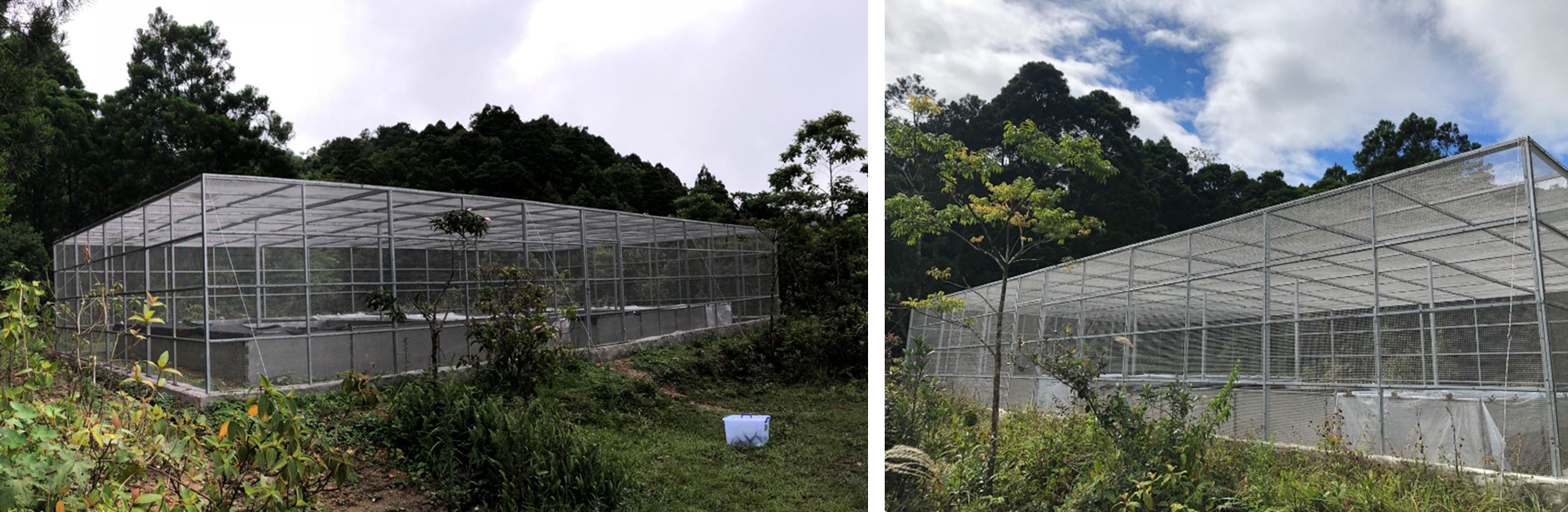
*

***Overview-Patches***

***
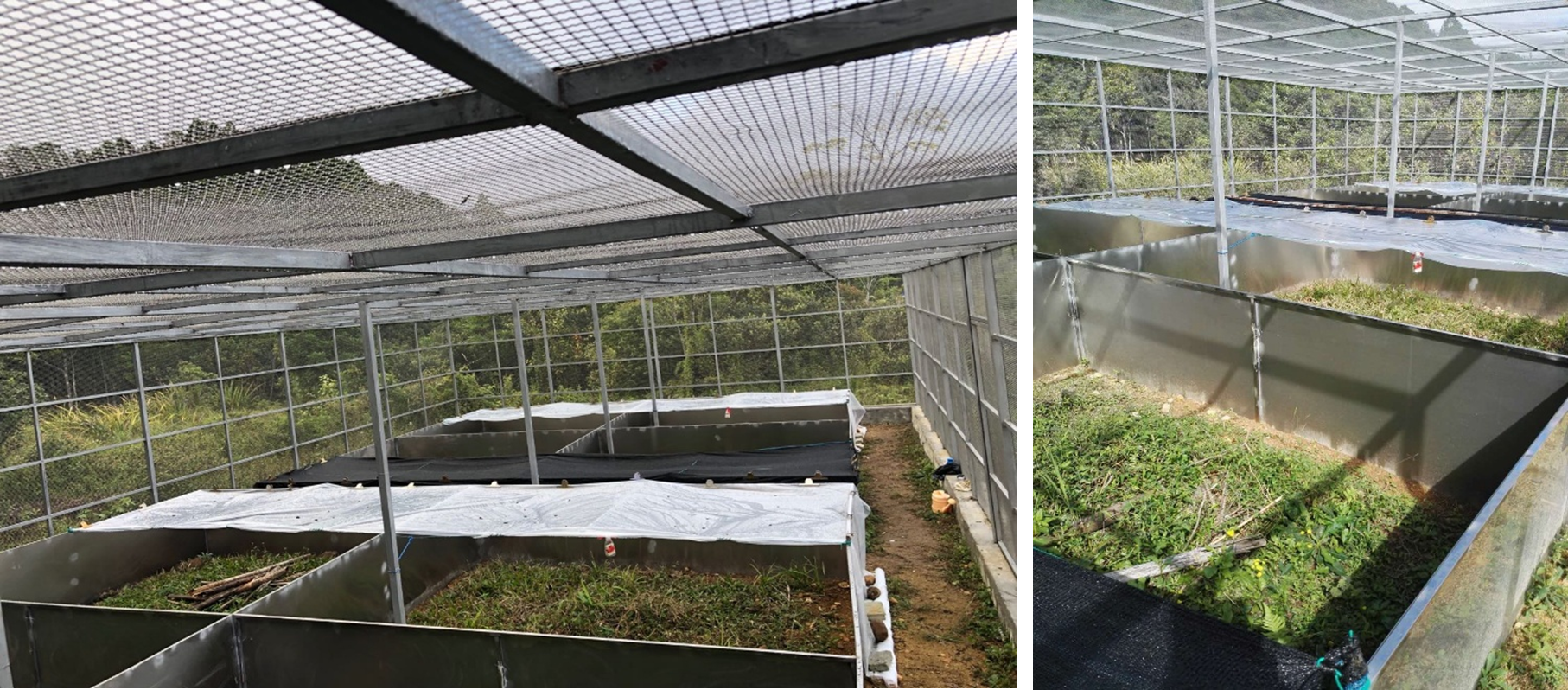
***
